# Incidence of pulmonary tuberculosis under the regular COVID-19 epidemic prevention and control in China

**DOI:** 10.1186/s12879-022-07620-y

**Published:** 2022-07-24

**Authors:** Ziwei Wu, Ziyi Chen, Siyu Long, Aiping Wu, Hongsheng Wang

**Affiliations:** 1 Institute of Dermatology, Chinese Academy of Medical Sciences & Peking Union Medical College, Nanjing, 210042 China; 2 Department of Health Toxicology, School of Public Health, Nanjing Medical University, Nanjing, 211166 China; 3 Center for Systems Medicine, Institute of Basic Medical Sciences, Chinese Academy of Medical Sciences & Peking Union Medical College, Beijing, 100005 China; 4 Suzhou Institute of Systems Medicine, Suzhou, 215123 Jiangsu China; 5 Center for Global Health, School of Public Health, Nanjing Medical University, Nanjing, China; 6 Jiangsu Key Laboratory of Molecular and Biology for Skin Diseases and STIs, Institute of Dermatology, Chinese Academy of Medical Sciences and Peking Union Medical College, Nanjing, China

**Keywords:** COVID-19, Intervention, Tuberculosis, Incidence, Model

## Abstract

**Background:**

The COVID-19 pandemic has driven public health intervention strategies, including keeping social distance, wearing masks in crowded places, and having good health habits, to prevent the transmission of the novel coronavirus (SARS-CoV-2). However, it is unknown whether the use of these intervention strategies influences morbidity in other human infectious diseases, such as tuberculosis.

**Methods:**

In this study, three prediction models were constructed to compare variations in PTB incidences after January 2020 without or with intervention includes strict and regular interventions, when the COVID-19 outbreak began in China. The non-interventional model was developed with an autoregressive integrated moving average (ARIMA) model that was trained with the monthly incidence of PTB in China from January 2005 to December 2019. The interventional model was established using an ARIMA model with a continuing intervention function that was trained with the monthly PTB incidence in China from January 2020 to December 2020.

**Results:**

Starting with the assumption that no COVID-19 outbreak had occurred in China, PTB incidence was predicted, and then the actual incidence was compared with the predicted incidence. A remarkable overall decline in PTB incidence from January 2020 to December 2020 was observed, which was likely due to the potential influence of intervention policies for COVID-19. If the same intervention strategy is applied for the next 2 years, the monthly PTB incidence would reduce on average by about 1.03 per 100,000 people each month compared with the incidence predicted by the non-interventional model. The annual incidence estimated 59.15 under regular intervention per 100,000 in 2021, and the value would decline to 50.65 with strict interventions.

**Conclusions:**

Our models quantified the potential knock-on effect on PTB incidence of the intervention strategy used to control the transmission of COVID-19 in China. Combined with the feasibility of the strategies, these results suggested that continuous regular interventions would play important roles in the future prevention and control of PTB.

**Supplementary Information:**

The online version contains supplementary material available at 10.1186/s12879-022-07620-y.

## Background

Tuberculosis (TB) is a serious threat to global public health, with about 10 million people suffering from pulmonary tuberculosis (PTB) and nearly 2 million people will die of this disease each year [1, 2]. Since 2007, PTB has become the leading cause of death from a single infectious agent [1]; despite the substantial achievements made under some expanded programs to strengthen the delivery of high-quality TB treatment [3–5] and improve the level of TB care and prevention [6, 7]. Further strengthening of efforts are required to provide better disease control. From 2000 to 2018, the average decline in TB incidence was 1.6% per year, and the cumulative reduction in TB incidence between 2015 and 2018 was only 6.3% [1]. In 2015, the World Health Organization launched the End TB Strategy to end the global TB epidemic by 2030 [8].

China, despite having achieved great progress in TB prevention and care over the past two decades [9], remains the second-largest contributor to the global burden of new TB cases, accounting for 8.5% of the global total, second only to India (26%) [1]. Moreover, TB incidence between 2015 and 2018 almost did not decrease in China, which is a cumulative reduction far below the average level worldwide. Multiple models have shown that, in addition to active case finding and effective treatment for an active case, prevention remains the key component of an intervention strategy [10–13]. However, existing intervention strategies for controlling TB, such as the enhancement of TB services, would be insufficient to eliminate TB [14]. Bacillus Calmette–Guérin (BCG), the only available TB vaccine, can only protect young children. BCG has been demonstrated to prevent severe extrapulmonary tuberculosis and also plays a weaker role in preventing PTB [15]. The pipeline for new TB-related diagnostics, drugs, and vaccines is progressing but at a slow pace [16]. Thus, new strategies must be developed to reduce TB incidence and mortality and fulfill the goals set in the End TB Strategy.

The urgent response mounted as a result of the COVID-19 outbreak caused by the severe acute respiratory syndrome coronavirus 2 (SARS-CoV-2) through social intervention strategies provide a perfect reference for improving the effectiveness of PTB prevention [17, 18]. By the end of 2020, the outbreak had resulted in over 83 million COVID-19 infections and over 1,800,000 deaths [19]. To contain the outbreak, China implemented unprecedented strict intervention strategies on 23 January 2020. The entire city of Wuhan was quarantined, strict measures limiting travel and public gatherings were introduced, public spaces were closed, rigorous temperature monitoring was implemented, and people were asked to maintain social distance, wear masks, and frequently wash hands nationwide. After over 2 months of unremitting efforts, the transmission of COVID-19 had been effectively controlled in China, and the lockdown in Wuhan was lifted on 8 April 2020 [20]. Nevertheless, scattered outbreaks of COVID-19 occurred in some areas, and cases imported from abroad were recorded. Accordingly, the same strategies, i.e., maintaining social distancing, wearing masks, and washing hands frequently, have been implemented as the regular COVID-19 epidemic prevention and control protocol. Interestingly, a remarkable decrease in PTB incidence in China was simultaneously observed during the COVID-19 outbreak. In addition, the PTB incidence in China had been effectively controlled under the regular COVID-19 epidemic prevention and control for the next 8 months. Thus, the interventional strategies conducted during the COVID-19 pandemic likely played a role in reducing PTB incidence as both diseases spread through the air.

The effectiveness of various measures to lower or control PTB incidence could potentially be fitted into an interventional model, which in turn could be further used to forecast future trends of PTB incidence according to previous data. To explore the availability of some COVID-19 interventional strategies, such as maintaining social distance, wearing masks, and regular handwashing, for the control of PTB incidence, we analyzed and estimated the observed impact of intervention effects on PTB incidence in China. Both a non-interventional model and an interventional model with different levels were constructed to predict the future development of PTB incidence. These results can guide reasonable policies for strengthening the control of PTB and other infectious diseases.

## Methods

### Data source

**Fig. 1:**
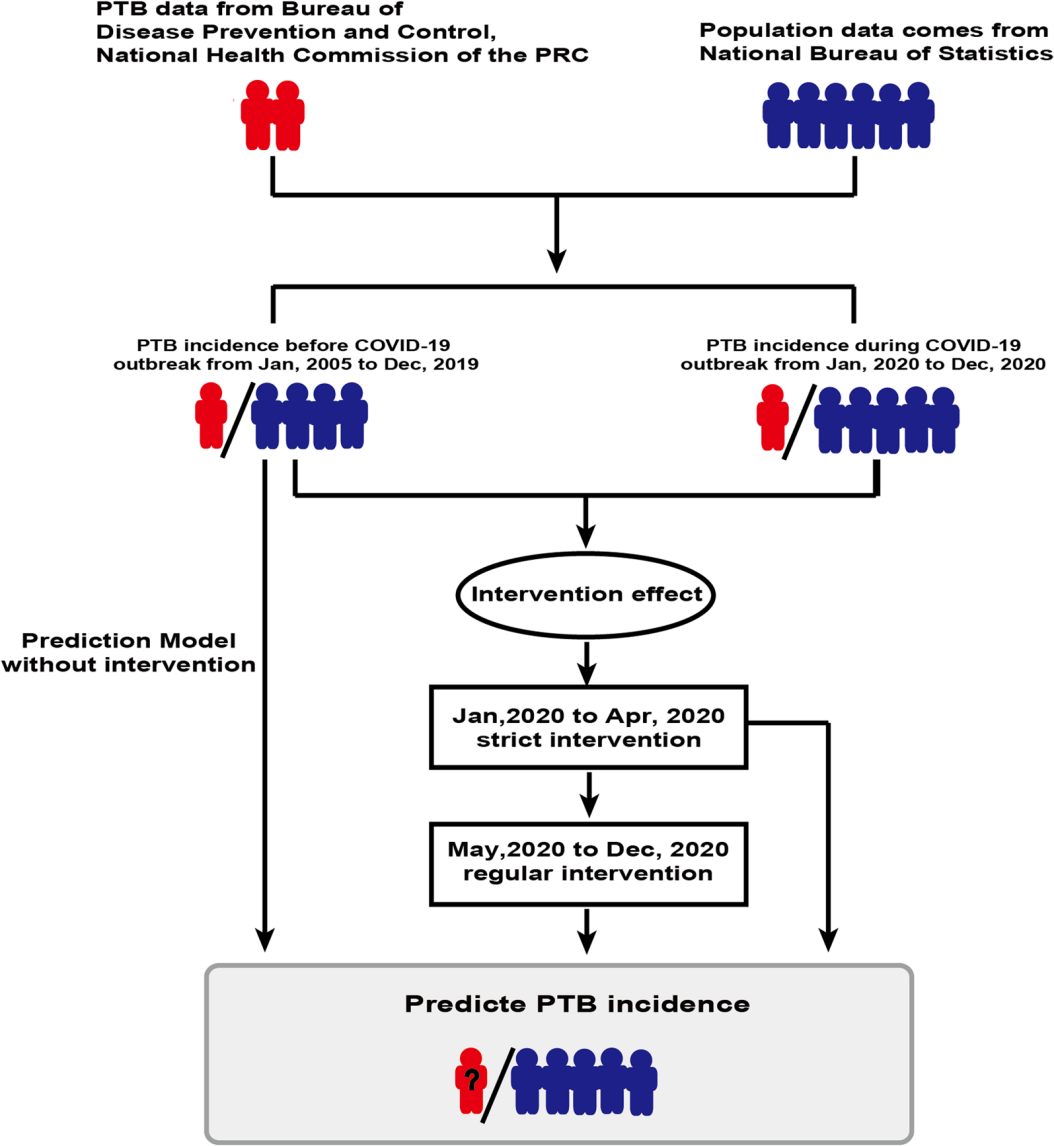
Overview of the study design. The monthly PTB incidence from January 2005 to December 2020 and the total population data reported at the end of each year from 2004 to 2019 were collected and used to calculate the monthly incidence between January 2005 to December 2020. The incidence data before the COVID-19 outbreak between January 2005 and December 2019 were then used to construct a prediction model without intervention, and the data both before and during the COVID-19 outbreak (between January 2020 and December 2020) were utilized to construct a prediction model under intervention

Data on monthly PTB incidence from January 2005 to December 2020 were collected from the National Statutory Infectious Disease Report Statistics Table of the Bureau of Disease Prevention and Control, National Health Commission of the People’s Republic of China (Fig. 1) [21]. The total population data reported at the end of each year from 2004 to 2019 were extracted from the National Bureau of Statistics (Fig. 1) [22]. Monthly PTB incidence was calculated by dividing the number of newly reported PTB cases by the total population number released at the end of the previous year. The monthly reported PTB cases exclude latent PTB [21], population numbers [22], and regular PTB incidences are listed in Additional file 3.

### Statistical methodology

#### Overview of model construction

In this study, monthly PTB incidence was used to evaluate the intervention effects of some intervention strategies conducted during the COVID-19 outbreak on the PTB epidemic and to predict the future tendency of PTB incidence (Fig. 1). With the different states of COVID-19 epidemic as the boundary, the time series data from January 2005 to December 2020 were divided into three parts, namely, data before the COVID-19 outbreak in China from January 2005 to December 2019, data during the implementation of strict interventions before the lockdown of Wuhan from January 2020 to April 2020, and data during the regular COVID-19 epidemic prevention and control from May 2020 to December 2020. Accordingly, three computational autoregressive integrated moving average models (ARIMA) were constructed to predict PTB incidence with or without intervention after the COVID-19 outbreak. First, using the data before the COVID-19 outbreak, a prediction model without intervention as a reference model was used to predict PTB incidence from January 2020 to December 2021. The indirect impact of intervention strategies for controlling COVID-19 on future PTB incidence was then modeled. A prediction model with the intervention was developed using the ARIMA model with a continuing intervention function to evaluate PTB incidence from May 2020 to December 2021. The degree of intervention was graded to establish a new intervention model on the basis of the above intervention model with the intervention, including strict interventions from January 2020 to April 2020 and regular interventions from May 2020 to December 2020. This new interventional model was used to predict PTB incidence from January 2021 to December 2022.

#### Construction of the non-interventional ARIMA model

The non-interventional model is a pure ARIMA model that uses only the time-series response data [23, 24]. The non-interventional ARIMA model was constructed in four steps (Additional files 1 and 2). First, non-stationary time series data were transformed into stationary time series by differencing processes. This step was essential to stabilize the time series data and reduce residuals. The augmented Dickey–Fuller (ADF) test was used to analyze the unit root for the input time series data suggested by the null hypothesis. Second, optimal model parameters were determined according to the autocorrelation function (ACF) and partial ACF (PACF). In general, more than one tentative model was chosen in this step for model identification and parameter estimation. Third, the model with the lowest Akaike information criterion and Schwarz criterion values was finally selected as the best ARIMA model [25]. The parameters were estimated using the maximum likelihood method to examine the residuals of the optimal model. The residual was set to be white noise to indicate that the model had completely extracted information from the original data. Moreover, the ACF and PACF plots of the residuals should have had no significant correlation [26–28]. Finally, the prediction ability of the model was evaluated. The PTB incidence data of the model in the last 15 months were fitted with this optimal ARIMA model. The prediction results were compared with criterion interval to evaluate the performance of forecasting [29, 30]. The model with the optimal accuracy was further used to predict the monthly incidence of PTB.

#### Construction of the interventional ARIMA model

The interventional ARIMA model with input series can be used to simulate and forecast the time series response and estimate the intervention effect [31]. Distinct from the non-interventional ARIMA model, a vector consisting of 0, 1, and 2 representing the inference time points was added as an input series to a transfer function. Specifically, a vector with a length of 180 representing each month from January 2005 to December 2020 was prepared. The months from January 2020 to April 2020 were marked as 2, due to being under strict intervention such as the implementation of travel bans and public gatherings restrictions, the closing of public spaces, and the practice of rigorous temperature monitoring in China. From May 2020, the months under regular intervention were marked as 1, during which people are asked to maintain social distance, wear masks, and wash hands frequently nationwide. Other time points were marked as 0. Similar to the AR part of the ARIMA model for the noise series, exponentially weighted and infinitely distributed lags were introduced into the transfer function. After model construction, the PTB incidence data from January 2014 to June 2020 were fitted with the optimized model. The predicted results were then compared with the actual observed data during this period. The model with the optimal accuracy was further used to predict the monthly incidence of PTB from July 2020 to December 2021.

### Data analysis

After constructing the non-interventional and interventional models as above, the future PTB incidence with or without the intervention strategies was then determined. The ARIMA non-interventional model constructed with the data from January 2005 to December 2019 was used to predict the potential PTB incidence if COVID-19 outbreak did not occur and thus no associated intervention was implemented. Via the non-interventional model, PTB incidence without any inference was predicted and used as the reference state. The ARIMA interventional model was then used to predict PTB incidence trends under strict interventions in the next few years by imposing a continuous intervention. Finally, the ARIMA interventional model with the level of intervention was utilized to predict PTB incidence trends under regular interventions in the next few years by imposing a continuous intervention.

## Results

### Characterization of PTB incidence before and after the COVID-19 outbreak

**Fig. 2:**
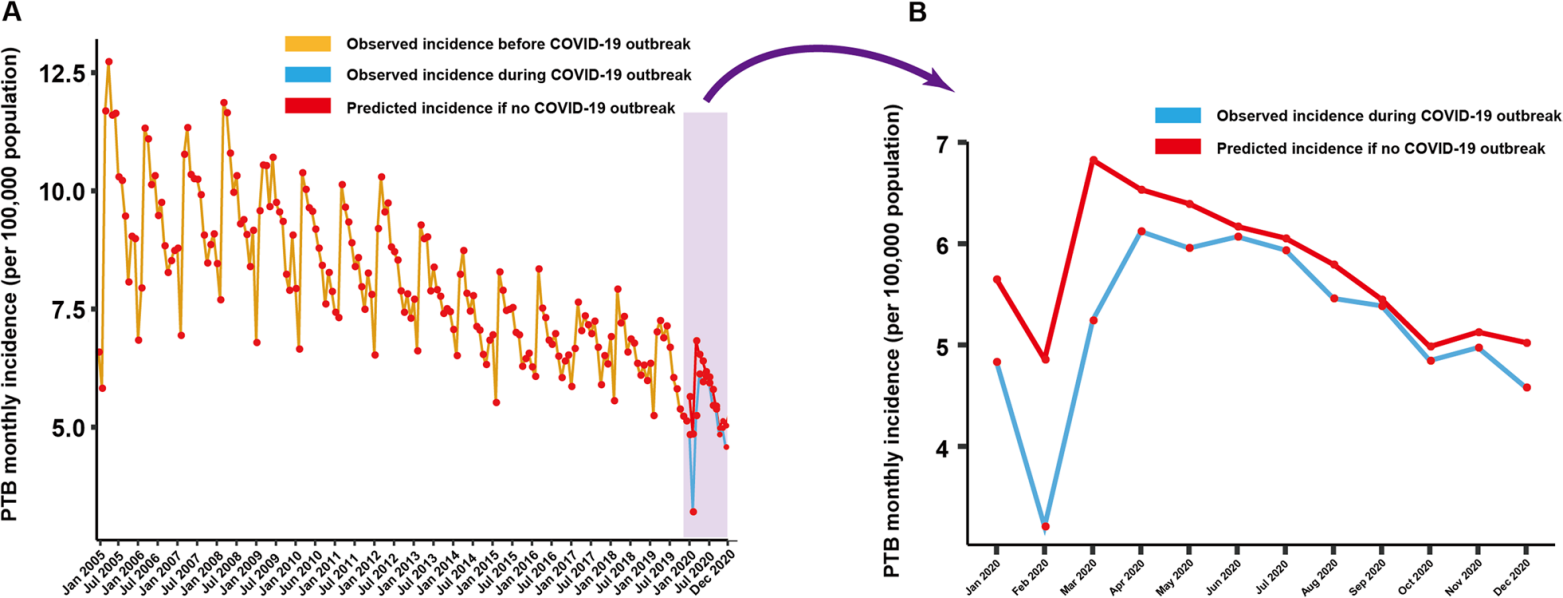
Incidence data estimated from the non-intervention model. **A** Time series of monthly PTB incidence from January 2005 to December 2020. The red line indicates the observed incidence before the COVID-19 outbreak. The sky blue line represents the observed incidence during the COVID-19 outbreak. The orange line denotes the incidence data between January 2020 and December 2020 as predicted from the non-intervention model. **B** The observed TB incidence (sky blue line) and the incidence predicted with the data before the COVID-19 outbreak (orange line) between January 2020 and December 2020

Before the COVID-19 outbreak, a stable and periodical cycle of annual PTB incidence was observed from January 2005 to December 2019 (Fig. 2A). The monthly incidence fluctuated from 5 to 13 per 100,000 every year. The highest incidence usually occurred in March or April and then it gradually decreased to the lowest value in February of the following year (Fig. 2A). Therefore, a dramatic rise in PTB incidence from February to March was observed in almost every year (Fig. 2A). The average increase in PTB incidence from February to March was 2.25, wherein the highest value was 5.87 in 2005 and the lowest value was 0.97 in 2009. Another slight increase in PTB incidence was usually observed from October to December each year. However, during the COVID-19 outbreak, PTB incidence sharply decreased when compared with the data from before the COVID-19 outbreak. As indicated in Fig. 2, the average monthly PTB incidence from the previous 6 years (from 2005 to 2019) were 7.10, 6.89, 9.58, 9.41, 8.96, 8.71, 8.49, 8.25, 7.83, 7.22, 7.45, and 7.52, respectively, whereas these numbers in 2020 decreased to 4.83, 3.21, 5.25, 6.12, 5.96, 6.07, 5.94, 5.46, 5.39, 4.85, 4.97, and 4.58, respectively. Similarly, the monthly PTB incidence in February was the lowest in 2020, although there was an overall decline.

### Predicted PTB incidence in 2020 with the non-interventional model

The remarkable overall decline in PTB incidence since the COVID-19 outbreak in China may be indirectly influenced by the drastic intervention measures enacted to contain the novel coronavirus. The exact inference of intervention strategies conducted during the COVID-19 outbreak was quantified by constructing the ARIMA non-interventional model to predict the reference PTB incidence in 2020. As shown in Fig. 2B, the incidence estimated with this prediction model was significantly higher than the actual observed incidence in each month. The predicted monthly PTB incidence from January 2020 to December 2020 was 5.65, 4.86, 6.82, 6.53, 6.39, 6.17, 6.05, 5.79, 5.45, 4.98, 5.13, and 5.02 respectively, whereas the actual observed value during these months was 4.83, 3.21, 5.25, 6.12, 5.96, 6.07, 5.94, 5.46, 5.39, 4.85, 4.97, and 4.58, respectively (Fig. 2A and Table 1). The difference between the predicted PTB incidence and the observed value ranged from 0.06 (September 2020) to 1.65 (February 2020) per 100,000.

**Table 1:** Predicted monthly PTB incidence in 2020 and 2021 under the presence or absence of a persistent intervention

	Observed incidence	No intervention (95% CI)		Strict intervention (95% CI)		Regular intervention (95% CI)
Month	2020	2020	2021	2020	2021	2021
1	4.834	5.650 (4.649, 6.650)	5.261 (3.974, 6.548)		4.127 (2.869, 5.385)	4.707 (3.677, 5.738)
2	3.209	4.857 (3.856, 5.858)	4.525 (3.224, 5.826)		3.093 (1.809, 4.378)	3.649 (2.613, 4.684)
3	5.245	6.825 (5.798, 7.851)	6.477 (5.150, 7.805)		5.073 (3.762, 6.383)	5.635 (4.568, 6.702)
4	6.120	6.534 (5.492, 7.575)	6.195 (4.844, 7.547)		5.212 (3.877, 6.548)	5.770 (4.679, 6.861)
5	5.956	6.394 (5.335, 7.453)	6.052 (4.677, 7.427)	5.475 (4.431, 6.520)	4.885 (3.405, 6.365)	5.617 (4.501, 6.733)
6	6.068	6.169 (5.093, 7.244)	5.833 (4.435, 7.231)	5.370 (4.322, 6.419)	4.707 (3.199, 6.215)	5.515 (4.375, 6.656)
7	5.936	6.054 (4.962, 7.146)	5.715 (4.294, 7.135)	5.224 (4.135, 6.312)	4.578 (3.027, 6.130)	5.393 (4.229, 6.557)
8	5.459	5.794 (4.686, 6.902)	5.450 (4.007, 6.893)	4.957 (3.840, 6.075)	4.313 (2.723, 5.904)	5.053 (3.865, 6.240)
9	5.386	5.449 (4.326, 6.573)	5.106 (3.641, 6.572)	4.615 (3.467, 5.762)	3.970 (2.341, 5.599)	4.807 (3.596, 6.017)
10	4.846	4.983 (3.844, 6.123)	4.641 (3.154, 6.128)	4.149 (2.973, 5.325)	3.504 (1.837, 5.171)	4.313 (3.080, 5.546)
11	4.974	5.127 (3.972, 6.282)	4.781 (3.273, 6.290)	4.285 (3.081, 5.489)	3.643 (1.939, 5.347)	4.450 (3.195, 5.705)
12	4.578	5.023 (3.853, 6.193)	4.677 (3.147, 6.206)	4.183 (2.951, 5.414)	3.540 (1.800, 5.280)	4.243 (2.966, 5.520)

Unit: 1/100,000

No intervention indicates the prediction model under no intervention. Persistent intervention is defined as the prediction model under a persistent intervention includes strict and regular interventions

### PTB incidence from 2020 to 2021 predicted with the interventional model under strict intervention conditions

**Fig. 3:**
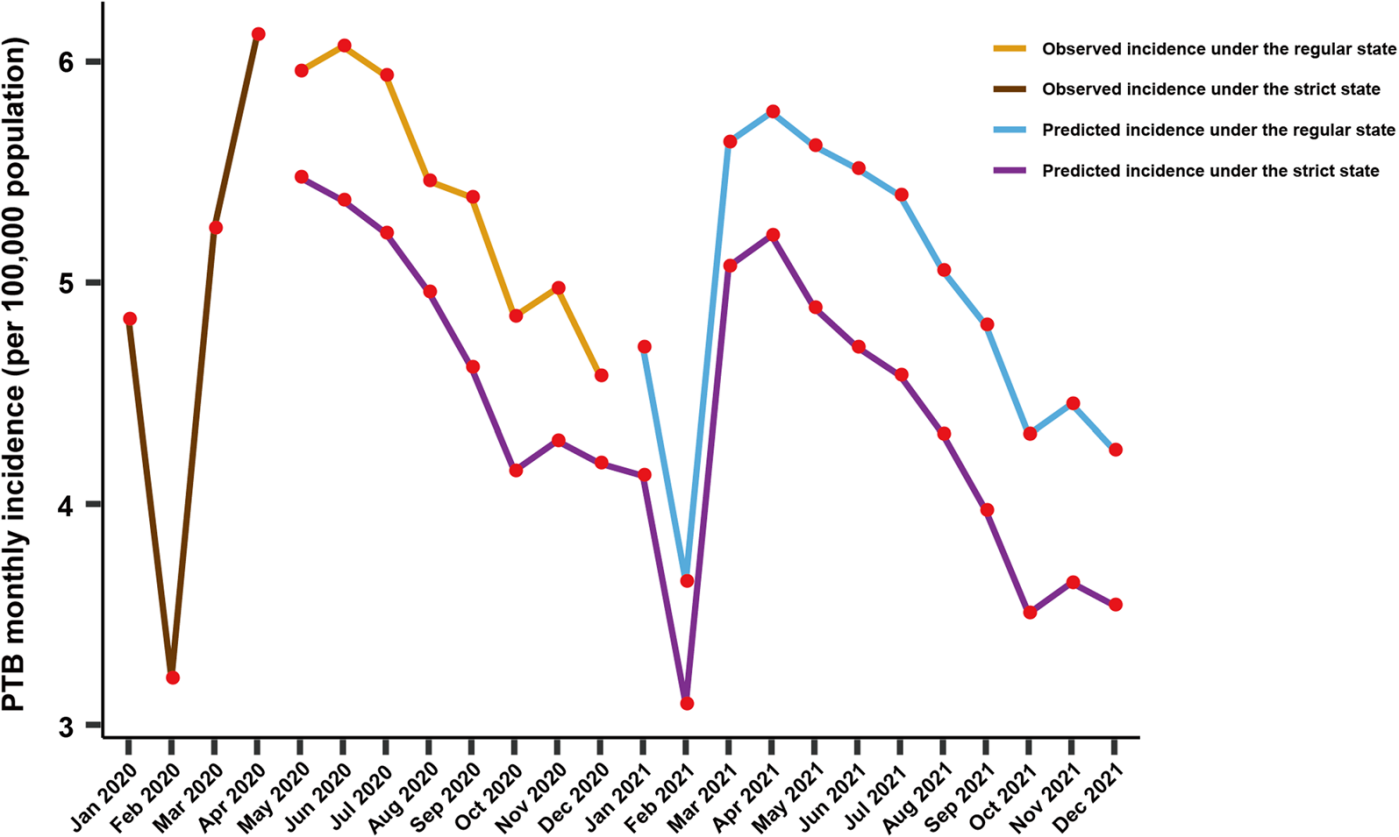
Evaluation of the intervention effect on the tendency of PTB incidence with both the strict and regular interventional models. Time series of PTB monthly incidence from January 2020 to December 2021. The brown line indicates the observed incidence under the strict state of COVID-19 epidemic prevention and control. The blue line represents the observed incidence under the regular state of COVID-19 epidemic prevention and control. The black line denotes the incidence data from May 2020 to December 2021 as predicted from the intervention model under strict intervention. The brown line denotes the incidence data from January 2021 to December 2021 as predicted from the intervention model under regular intervention

The significant decline in PTB incidence observed since the implementation of the strict intervention during the COVID-19 outbreak from January 2020 to April 2020 in China highlighted the importance of strict social interventions in preventing PTB. Therefore, the ARIMA interventional model was constructed with the incidence data from January 2005 to April 2020 to speculate the future tendency of PTB incidence under strict interventions. As illustrated in Fig. 3, the incidence predicted for the next 2 years would stay at a relatively low level when the strict interventional measures are maintained. The monthly incidence would be reduced by 1.03 per 100,000 in every month compared with those without intervention before the COVID-19 outbreak (Fig. 3 and Table 1). The mean incidence from 2020 to 2021 predicted with this interventional model would decline to 4.51 per 100,000.

### PTB incidence from 2020 to 2021 predicted with the interventional model under regular intervention conditions

Since the COVID-19 epidemic had gone into remission in April 2020 in China, prevention and control strategies had also been adjusted as regular state, which continues to this day. These regular intervention strategies were tested by comparing the predicted PTB incidence under different intervention strategies. As illustrated in Fig. 3, the incidence predicted for the next year would stay at a relatively low level when the regular interventional measures are maintained. The annual incidence estimated 59.15 under regular intervention per 100,000 in 2021, and the value would decline to 50.65 with strict interventions. The monthly incidence predicted with the intervention model under regular intervention conditions would be generally larger than that under strict intervention conditions. Moreover, the difference among the annual incidence in 2021 of the two groups was statistically significant. The preventive effect on PTB incidence would clearly be better under a strict intervention strategy. In theory, a strict intervention is difficult to implement continuously. Thus, regular intervention strategies are more conducive to promotion and implementation, which would represent a huge achievement for PTB prevention.

### Model prediction effect evaluation

**Fig. 4:**
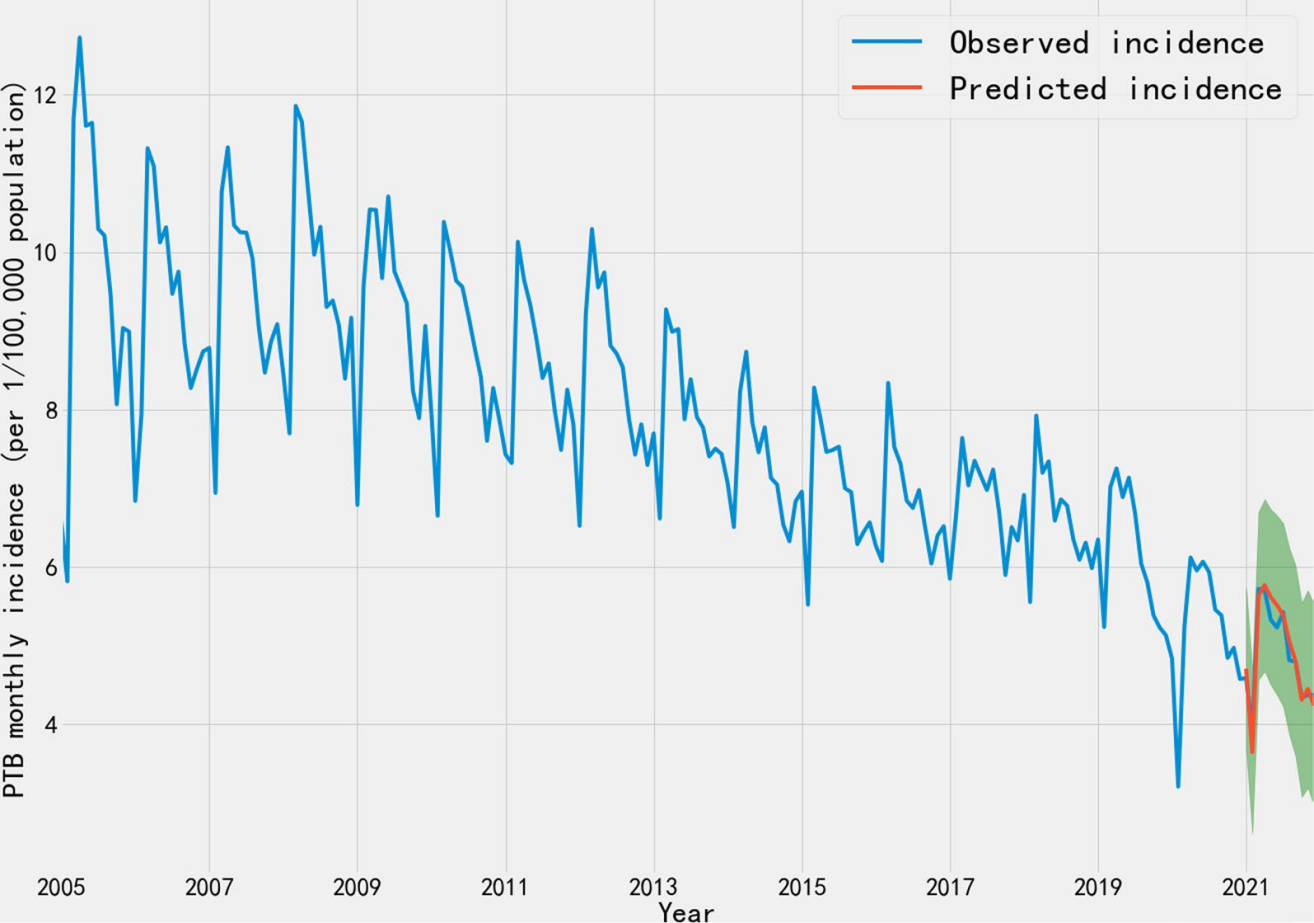
Predicted and actual monthly PTB incidence in China from January 2005 to December 2021. Time series of monthly PTB incidence from January 2005 to December 2021. The blue line indicates the observed incidence. The red line denotes the incidence data between January 2021 and December 2021 as predicted from the intervention model. The green interval represents the a time series forecast prediction interval (95%) for the predictions

Until December 31, 2021, China has continued to implem*e*nt regular interventions for COVID-19. So we compared the predicted values and actual incidence which gained in 2021 to validate the accuracy of intervention model under regular intervention conditions. Figure 4 showed that the predicted values in the monthly incidence of Chinese PTB is generally within the 95% *CI*, indicating that the model has good prediction performance. Meanwhile, it can be found that the trend predicted by the model is consistent with the actual trend from January 2005 to December 2021, and the incidence of PTB in China continued to decrease in 2021.

## Discussion

COVID-19 is primarily a respiratory disease, and its causal virus (SARS-CoV-2) is mainly transmitted between people via respiratory droplets and contact routes. Limiting close contact between infected people and others is central to breaking the transmission of the virus [32]. Since January 2020, some of the strict strategies to prevent COVID-19 and reduce its spread in the public include wearing masks, living in rooms with good ventilation, having good hand hygiene, keeping physical distance, and avoiding crowded indoor gatherings. With strong government intervention, the COVID-19 outbreak has been well controlled in China [33], and the regular COVID-19 epidemic prevention and control protocol has been implemented since April 2020. Clinicians and researchers can apply knowledge from experiences with effective prevention and control of COVID-19 to prevent other infectious diseases, especially respiratory diseases. For example, the ultimate goal of public health interventions should be to reduce PTB burden through early detection and disruption of the chain of transmission [34]. Unfortunately, a study in China estimated that the current strategy has had a limited impact on the reduction of PTB incidence and mortality [12]. Nevertheless, as of June 2020, the average monthly mortality due to PTB in 2020 has dropped by 32.43% compared with that in the past 5 years in China [21]. In theory, effective strategies, such as maintaining social distance, wearing masks, and regular handwashing to prevent COVID-19, which are based on limiting close contact between infected people and uninfected people, could be helpful to control the spread of respiratory infectious diseases. Moreover, no definitive quantitative studies have been conducted to systematically assess the effects of other respiratory diseases that are transmitted from humans to humans via respiratory droplets and air, such as PTB. Our study provides a good avenue to quantify the potential effects of intervention strategies for preventing COVID-19 on PTB incidence. The modeling results indicated that if the implementation of some of the aforementioned measures are continued post-COVID-19, there may be positive effects in preventing other infectious diseases, such as PTB.

The potential effects of various intervention strategies on PTB were quantified using the ARIMA model, which is the most common time-series prediction model in statistical modeling. The ARIMA model has also been previously used in the field of infectious diseases [35, 36]. However, when the linear time series under study is disturbed by some external events known as an intervention, the forecasting performance of the ARIMA model may be affected. Model performance after such a disturbance can be improved by employing appropriate techniques, such as ARIMA intervention modeling. Intervention modeling is utilized to account for the impact of any unprecedented events in the time-series data. In this study, two models, namely, non-intervention and interventional models, were constructed to evaluate the impact of control measures implemented during the COVID-19 outbreak on PTB. If there had been no COVID-19 outbreak in China, according to the ARIMA model, PTB incidence from January 2020 to December 2020 would have been significantly higher than the actual PTB incidence observed during the COVID-19 outbreak (Table 1 and Additional file 3). These findings might be explained by the positive effect of China’s intervention strategies for stopping the public spread of SARS-CoV-2 on breaking the chains of transmission of Mycobacterium tuberculosis. Although the PTB incidence has been on a decreasing trend year by year in recent years and reached the lowest level in history in 2021 (Fig. 4), assuming China maintained these strict intervention strategies in 2020 and 2021, monthly PTB incidence would decrease at an average of 1.03 per 100,000 each month compared with the absence of interventions. Meanwhile, the annual incidence estimated with the non-interventional model was 64.71 per 100,000 in 2021, the annual incidence was 59.15 with the interventional model under regular intervention conditions, and the value would decline to 50.65 with the interventional model under strict intervention conditions. Considering that strict intervention is difficult to implement continuously, the regular intervention strategies are more conducive to promotion and implementation.

This study has several limitations. First, the analysis was based on the ARIMA model that was fitted with the epidemic data in China only. The model and the results must be validated with further prospective studies using large cohorts. Secondly, the incubation period for PTB is about three months or even longer, intervention strategies may cause the symptoms to appear later. Therefore, the prediction results should be comprehensively considered according to the actual situation. Finally, this study focused on the mixed effects of intervention measures. However, many confounding factors could have contributed to the reduction in PTB incidence. For example, recent research suggests that the COVID-19 pandemic has dramatically impacted TB diagnosis and case finding and has artificially decreased the number of TB cases in some countries including India, a country with a large number of PTB cases [22, 37, 38]. Therefore, the PTB cases used in this research were a measure of diagnosis instead of actual TB burden. Thus, these interventional measures would only affect those who would have had contracted PTB without the intervention, and the intervention is not effective for the treatment of patients with tuberculosis and for the prevention of reinfection of those with reactivation [39, 40].

Identifying any single-factor effect on preventing the development of the TB epidemic in China is challenging. Therefore, additional efforts are warranted to precisely evaluate the prevention effects of COVID-19-related social interventions on tuberculosis in China and other countries.

Interruption in the transmission of TB remains an important concern in China, a country with a high TB burden. Our data and modeling results suggested that the regular strategies implemented to control COVID-19 have also helped control the incidence of tuberculosis in China.

In summary, our findings have important implications for clinical and public health policies for tuberculosis prevention via the disruption of the chain of tuberculosis transmission. We recommend several strategies for the regular prevention of TB, including wearing of masks in endemic regions, provision of government-subsidized masks for crowded public places, maintaining good hand hygiene, avoiding large crowded indoor gatherings, and controlling the number of people in gatherings.

## Conclusions

With the ARIMA prediction model, the knock-on effects of intervention strategies for COVID-19 on PTB incidence were successfully estimated. If the same intervention strategy for controlling the spread of COVID-19 were maintained in 2020, the monthly PTB incidence would have decreased on average by about 1.03 per 100,000 people each month compared with the incidence predicted by the non-interventional model. The annual incidence estimated without intervention was 64.71 per 100,000 in 2021, and the annual incidence was 59.15 under regular intervention. This value would decline to 50.65 with strict intervention. Combined with the feasibility of these strategies, these results suggested that continuous regular interventions may play important roles in the future prevention and control of PTB.

## Supplementary Information

**Additional file 1.** Introduction to ARIMA Model.

**Additional file 2.** Jupyter (Python) code used for the ARIMA analysis.

**Additional file 3.** The monthly reported PTB cases, total population numbers per year and the normalized PTB incidences from January 2014 to December 2020 in China.

## Data Availability

The datasets generated and/or analysed during the current study are available from the corresponding author on reasonable request.

## Abbreviations

TB: Tuberculosis
PTB: Pulmonary tuberculosis
BCG: Bacillus Calmette–Guérin
SARS-CoV-2: Syndrome coronavirus 2
ACF: Autocorrelation function
PACF: Partial ACF
