# Supplementary material for: Incidence of pulmonary tuberculosis under the regular COVID-19 epidemic prevention and control in China
Source: BMC Infect Dis. 2022 Jul 24;22:641. doi: 10.1186/s12879-022-07620-y (PMC9308895; doi:10.1186/s12879-022-07620-y)
Supplement: Supplementary file 1 — Additional file 1. Introduction to ARIMA Model. [file 12879_2022_7620_MOESM1_ESM.doc]

**Additional file 1: Introduction to ARIMA Model**

***General overview of ARIMA***

The ARIMA model is widely used in the areas of non-stationary time series forecasting and can be written as:

*ϕ*(*B*)(1−*B*)*^d^* *X_t_* = *θ(B)_ε t_*,

where *X_t_* represents a non-stationary time series at time t; *ε_t_* is white noise (zero mean and constant variance); d is the order of differencing; *B* is a backward shift operator defined by *BX_t_* = *X_t_*−1; *φ*(*B*) is the autoregressive operator, which can be defined as *ϕ*(*B*)=1−*ϕ*_1_ *B*−*ϕ*_2_*B*^2^ −⋯−*ϕ_p_B^p^*; and *θ*(*B*) is the moving average operator defined as *θ*(*B*)=1−*θ*_1_*B*−*θ*_2_*B*^2^ −⋯−*θ_q_B^q^*.

Generally, ARIMA models can be classified into five major groups, namely, AR (P) model; MA (q); ARMA (p, q); ARIMA (p, d, q); and ARIMA (p, d, q) × (P, D, Q)s. ARIMA (p, d, q) × (P, D, Q)s can combine both non-seasonal parts and seasonal factors of the time series data, i.e. the regular components and the seasonal components.

The regular components include the non-seasonal autoregressive (p), non-seasonal moving average (q), and non-seasonal order of differencing (d). The seasonal components include the seasonal autoregressive (P), seasonal moving average (Q), and non-seasonal order of differencing (D), as well as the seasonal period, e.g., s = 12 for monthly series (s). It has been shown that a complicated seasonal effect exists in the incidence of [pulmonary](../../../../C:/Users/pca/AppData/Local/youdao/dict/Application/8.10.3.0/resultui/html/index.html#/javascript:;) tuberculosis (PTB). Therefore, ARIMA (p, d, q) × (P, D, Q)s is often used in the study to predict the epidemic trend of infectious diseases. The model expression was defined as:


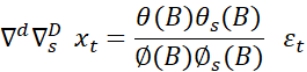


***ARIMA model with intervention analysis***

As discussed by Box and Jenkins, the general form of an intervention model can be written as:

*Y_t_* = *X_t_* + *Z_t_*,
where *X_t_* is the underlying intervention-free series usually modeled by ARIMA using pre-intervention series, and *Z_t_* is the change to the mean that is attributable to the intervention.

***Forms of intervening variables***

First, according to the types of intervention, the intervention variables can be classified into two major forms, namely, continuing interventions and impulse interventions.

For continuing interventions, a persistent intervention effect existed. The input variable was 0 before the intervention and 1 after the intervention. These kinds of intervention variables are called step functions.

In impulse interventions, a transient intervention occurred at a certain time series T, and the resulting intervention effect had an impact on this time period only. The input variable was 1 during the inference time and 0 at other time points. Intervention variables of this kind are called pulse functions.

Moreover, considering the modes of intervention, there are four basic forms of intervention models according to their influence on the data:

1. Form 1: There is a permanent constant change to the mean level. The intervention event starts at time T, and the intervention impact shall be maintained for some time.
2. Form 2: The intervention event occurred at time T and had a gradual increase and further leveled off at one point.
3. Form 3: A brief and constant intervention appeared during some time period. The intervention was limited to a temporary period of time and would not have much impact on other time points.
4. Form 4: The intervention impact peaked at time T and then gradually decreased to a lower level.

Considering the continued interference strategies conducted during the COVID-19 outbreak in China, Form 2 can represent the intervention pattern of this interference effect on the incidence of PTB.
