# Supplementary material for: Incidence of pulmonary tuberculosis under the regular COVID-19 epidemic prevention and control in China
Source: BMC Infect Dis. 2022 Jul 24;22:641. doi: 10.1186/s12879-022-07620-y (PMC9308895; doi:10.1186/s12879-022-07620-y)
Supplement: Supplementary file 2 — Additional file 2. Jupyter (Python) code used for the ARIMA analysis. [file 12879_2022_7620_MOESM2_ESM.doc]

**Additional file 2: Jupyter (Python) code used for the ARIMA analysis**

import numpy as np

import pandas as pd

import matplotlib.pyplot as plt

import matplotlib

plt.style.use('fivethirtyeight')

from matplotlib.pylab import rcParams

rcParams['figure.figsize'] = 28, 18

import statsmodels.api as sm

from statsmodels.tsa.stattools import adfuller

from statsmodels.tsa.seasonal import seasonal_decompose

import itertools

import warnings

warnings.filterwarnings("ignore")

plt.rcParams['font.sans-serif'] = ['SimHei']

matplotlib.rcParams['axes.unicode_minus'] =False

#### PTB INCIDENCE DATA ####

dateparse = lambda x: pd.to_datetime(x, format='%Y-%m', errors = 'coerce')

df = pd.read_excel(r"rate.xlsx",sheet_name='Sheet1')

df['date'] = df.apply(lambda x:(str(int(x['year'])) + '-' + str(int(x['month']))),axis=1).tolist()

df = df[['date','incidence','year','month','intervention']]

#Predicting with data from January 2005 to December 2019

df = df.iloc[:180,:2]

df = df[pd.Series(pd.to_datetime(df.index, errors='coerce')).notnull().values]

df.dropna(inplace=True)

df.to_excel('rate1.xlsx',index=None)

#Predicting with data from January 2005 to April 2020

df = df.iloc[:184,:2]

df = df[pd.Series(pd.to_datetime(df.index, errors='coerce')).notnull().values]

df.dropna(inplace=True)

df.to_excel('rate1.xlsx',index=None)

#Predicting with data from January 2005 to December 2020

df = df.iloc[:,:2]

df = df[pd.Series(pd.to_datetime(df.index, errors='coerce')).notnull().values]

df.dropna(inplace=True)

df.to_excel('rate1.xlsx',index=None)

df = pd.read_excel("rate1.xlsx",sheet_name='Sheet1', parse_dates=['date'], index_col='date', date_parser=dateparse)

## Identify the stability of the sequence

## Decompose trend, seasonal and random effects

## df = pd.DataFrame(df)

decomposition = seasonal_decompose(df)

trend = decomposition.trend # trend effects

seasonal = decomposition.seasonal # seasonal effects

residual = decomposition.resid # random effects

plt.subplot(411)

plt.plot(df, label=u'original series')

plt.legend(loc='best')

plt.subplot(412)

plt.plot(trend, label=u'trend cycle')

plt.legend(loc='best')

plt.subplot(413)

plt.plot(seasonal,label=u'seasonality')

plt.legend(loc='best')

plt.subplot(414)

plt.plot(residual, label=u'residual')

plt.legend(loc='best')

plt.tight_layout()

def TestStationaryPlot(ts):

rol_mean = ts.rolling(window = 12, center = False).mean()

rol_std = ts.rolling(window = 12, center = False).std()

plt.plot(ts, color = 'blue',label = u'original series')

plt.plot(rol_mean, color = 'red', linestyle='-.', label = u'Moving Average')

plt.plot(rol_std, color ='black', linestyle='--', label = u'Standard Deviation')

plt.xticks(fontsize = 25)

plt.yticks(fontsize = 25)

plt.xlabel(u'time（year）', fontsize = 25)

plt.ylabel(u'incidence', fontsize = 25)

plt.legend(loc='best', fontsize = 18)

plt.title(u'Moving Average and Standard Deviation', fontsize = 27)

plt.show(block= True)

def TestStationaryAdfuller(ts, cutoff = 0.01):

ts_test = adfuller(ts, autolag = 'AIC')

ts_test_output = pd.Series(ts_test[0:4], index=['Test Statistic','p-value','#Lags Used','Number of Observations Used'])

for key,value in ts_test[4].items():

ts_test_output['Critical Value (%s)'%key] = value

print(ts_test_output)

if ts_test[1] <= cutoff:

print(u"Reject the null hypothesis, data series have no unit root and is stationary.")

else:

print(u"Fail to reject the null hypothesis, data series have a unit root and is non-stationary.")

TestStationaryPlot(df)

TestStationaryAdfuller(df)

##Eliminate trend and seasonality

df_first_difference = df - df.shift(1)

df_seasonal_first_difference = df_first_difference - df_first_difference.shift(12) TestStationaryPlot(df_seasonal_first_difference.dropna(inplace=False))

TestStationaryAdfuller(df_seasonal_first_difference.dropna(inplace=False))

##White Noise Test

df_seasonal_first_difference.dropna(inplace = True)

r,q,p = sm.tsa.acf(df_seasonal_first_difference.values.squeeze(), qstat=True)

data = np.c_[range(1,41), r[1:], q, p]

table = pd.DataFrame(data, columns=['lag', "AC", "Q", "Prob(>Q)"])

print(table.set_index('lag'))

##Model ordering

p = d = q = range(0, 2)

pdq = list(itertools.product(p, d, q))

pdq_x_PDQs = [(x[0], x[1], x[2], 12) for x in list(itertools.product(p, d, q))]

a=[]

b=[]

c=[]

wf=pd.DataFrame()

for param in pdq:

for seasonal_param in pdq_x_PDQs:

try:

mod = sm.tsa.statespace.SARIMAX(df,order=param,seasonal_order=seasonal_param,enforce_stationarity=False,enforce_invertibility=False)

results = mod.fit()

print('ARIMA{}x{} - AIC:{}'.format(param, seasonal_param, results.aic))

a.append(param)

b.append(seasonal_param)

c.append(results.aic)

except:

continue

wf['pdq']=a

wf['pdq_x_PDQs']=b

wf['aic']=c

print(wf[wf['aic']==wf['aic'].min()])

##Model building

mod = sm.tsa.statespace.SARIMAX(df,

order=(1,1,1),

seasonal_order=(1,1,1,12),

enforce_stationarity=False,

enforce_invertibility=False)

results = mod.fit()

print(results.summary())

## Model checking

## Model diagnosis

results.plot_diagnostics(figsize=(15, 12))

plt.show()

## LB test

r,q,p = sm.tsa.acf(results.resid.values.squeeze(), qstat=True)

data = np.c_[range(1,41), r[1:], q, p]

table = pd.DataFrame(data, columns=['lag', "AC", "Q", "Prob(>Q)"])

print(table.set_index('lag'))

## Model prediction

# Static prediction

pred = results.get_prediction(start = 132, dynamic=False)

pred_ci = pred.conf_int()

df_forecast = pred.predicted_mean

df_truth = df['2016-1':]

df_pred_concat = pd.concat([df_truth, df_forecast,pred_ci],axis=1)

df_pred_concat.columns = [u'Original value',u'Predictive value',u'Lower limit',u'Upper limit']

df_pred_concat.head(60)

df_forecast = np.array(df_forecast)

df_truth = np.array(df_truth)

ax = df['2005-1':].plot(label='observed')

pred.predicted_mean.plot(ax=ax, label='One-step ahead forecast', alpha=.7)

ax.fill_between(pred_ci.index,pred_ci.iloc[:,0],pred_ci.iloc[:,1], color='r', alpha=.5)

plt.xticks(fontsize = 25)

plt.yticks(fontsize = 25)

ax.set_xlabel(u'time(year)')

ax.set_ylabel('incidence')

plt.legend()

plt.show()

# Calculating MSE and RMSE

mse = ((df_forecast - df_truth) ** 2).mean()

print(u'Mean Square Error of Predicted Value(MSE)is{}'.format(round(mse, 2)))

cc= np.sqrt(np.sum((df_forecast-df_truth)**2)/len(df_forecast))

print('Root Mean Square Error of Predicted Value(RMSE)is: ',cc)

# Dynamic prediction

pred_dynamic = results.get_prediction(start=pd.to_datetime('2016-1'), dynamic=True, full_results=True)

pred_dynamic_ci = pred_dynamic.conf_int()

df_forecast = pred_dynamic.predicted_mean

df_orginal = df['2016-1':]

df_pred_concat = pd.concat([df_orginal, df_forecast,pred_ci],axis=1)

df_pred_concat.columns = [u'Original value',u'Predictive value',u'Lower limit',u'Upper limit']

df_pred_concat.head(60)

ax = df['2005-1':].plot(label='observed', figsize=(20, 15))

pred_dynamic.predicted_mean.plot(label='Dynamic Forecast', ax=ax)

ax.fill_between(pred_dynamic_ci.index,pred_dynamic_ci.iloc[:, 0],pred_dynamic_ci.iloc[:, 1],color='r',alpha=.3)

ax.fill_betweenx(ax.get_ylim(),pd.to_datetime('2005-1'),df.index[-1],alpha=.1, zorder=-1)

plt.xticks(fontsize = 25)

plt.yticks(fontsize = 25)

ax.set_xlabel(u'time(year)',fontsize=25)

ax.set_ylabel(u'incidence',fontsize=25)

plt.legend(loc = 'upper left',fontsize=20)

plt.show()

# Calculating MSE and RMSE

df_forecast = np.array(df_forecast)

df_orginal = np.array(df_orginal)

mse = ((df_forecast - df_orginal) ** 2).mean()

print(u'Mean Square Error of Predicted Value(MSE)is{}'.format(round(mse, 2)))

cc= np.mean(np.sqrt(sum((df_forecast-df_orginal)**2)/len(df_forecast)))

print('Root Mean Square Error of Predicted Value(RMSE)is: ',cc)

# Forecast data for the next 10 years

forecast = results.get_forecast(steps=24)

# Get the confidence interval of the prediction

forecast_ci = forecast.conf_int()

df_forecast = forecast.predicted_mean

df_pred_concat = pd.concat([df_forecast,forecast_ci],axis=1)

df_pred_concat.columns = [u'Predictive value',u'Lower limit',u'Upper limit']

df_pred_concat.head(24)

#Draw a time series graph

ax = df.plot(label='observed', figsize=(20, 15))

forecast.predicted_mean.plot(ax=ax, label='Forecast')

ax.fill_between(forecast_ci.index,

forecast_ci.iloc[:, 0],

forecast_ci.iloc[:, 1], color='g', alpha=.4)

plt.xticks(fontsize = 20)

plt.yticks(fontsize = 18)

ax.set_xlabel('time(year)',fontsize=18)

ax.set_ylabel('incidence',fontsize=18)

plt.legend(loc = 'upper left',fontsize=20)

plt.show()
