# Supplementary material for: Incidence of pulmonary tuberculosis under the regular COVID-19 epidemic prevention and control in China
Source: BMC Infect Dis. 2022 Jul 24;22:641. doi: 10.1186/s12879-022-07620-y (PMC9308895; doi:10.1186/s12879-022-07620-y)
Supplement: Supplementary file 3 — Additional file 3. The monthly reported PTB cases, total population numbers per year and the normalized PTB incidences from January 2014 to December 2020 in China. [file 12879_2022_7620_MOESM3_ESM.doc]

**Additional file 3: The monthly reported PTB cases, total population numbers per year and the normalized PTB incidences** from January 2014 to December 2020 in China.

|  | **2005** | | | **2006** | | | **2007** | | | **2008** | | |
| --- | --- | --- | --- | --- | --- | --- | --- | --- | --- | --- | --- | --- |
| Month | Disease cases | Population/100,000 | incidence per 100, 000 | Disease cases | Population/100,000 | incidence per 100, 000 | Disease cases | Population/100,000 | incidence per 100, 000 | Disease cases | Population/100,000 | incidence per 100, 000 |
| **1** | 85577 | 129988 | 6.583 | 89436 | 130756 | 6.840 | 115457 | 131448 | 8.783 | 111688 | 132129 | 8.453 |
| **2** | 75625 | 129988 | 5.818 | 103823 | 130756 | 7.940 | 91235 | 131448 | 6.941 | 101689 | 132129 | 7.696 |
| **3** | 151935 | 129988 | 11.688 | 147996 | 130756 | 11.318 | 141508 | 131448 | 10.765 | 156679 | 132129 | 11.858 |
| **4** | 165419 | 129988 | 12.726 | 145029 | 130756 | 11.092 | 148930 | 131448 | 11.330 | 153978 | 132129 | 11.654 |
| **5** | 150823 | 129988 | 11.603 | 132367 | 130756 | 10.123 | 135933 | 131448 | 10.341 | 142612 | 132129 | 10.793 |
| **6** | 151330 | 129988 | 11.642 | 134879 | 130756 | 10.315 | 134775 | 131448 | 10.253 | 131699 | 132129 | 9.967 |
| **7** | 133807 | 129988 | 10.294 | 123829 | 130756 | 9.470 | 134695 | 131448 | 10.247 | 136378 | 132129 | 10.322 |
| **8** | 132729 | 129988 | 10.211 | 127509 | 130756 | 9.752 | 130404 | 131448 | 9.921 | 122923 | 132129 | 9.303 |
| **9** | 123062 | 129988 | 9.467 | 115540 | 130756 | 8.836 | 119078 | 131448 | 9.059 | 123998 | 132129 | 9.385 |
| **10** | 104851 | 129988 | 8.066 | 108177 | 130756 | 8.273 | 111325 | 131448 | 8.469 | 119821 | 132129 | 9.068 |
| **11** | 117468 | 129988 | 9.037 | 111384 | 130756 | 8.518 | 116503 | 131448 | 8.863 | 110896 | 132129 | 8.393 |
| **12** | 116859 | 129988 | 8.990 | 114262 | 130756 | 8.739 | 119421 | 131448 | 9.085 | 121114 | 132129 | 9.166 |

|  | **2009** | | | **2010** | | | **2011** | | | **2012** | | |
| --- | --- | --- | --- | --- | --- | --- | --- | --- | --- | --- | --- | --- |
| Month | Disease cases | Population/100,000 | incidence per 100, 000 | Disease cases | Population/100,000 | incidence per 100, 000 | Disease cases | Population/100,000 | incidence per 100, 000 | Disease cases | Population/100,000 | incidence per 100, 000 |
| **1** | 90160 | 132802 | 6.789 | 105877 | 133450 | 7.934 | 99617 | 134091 | 7.429 | 87893 | 134735 | 6.523 |
| **2** | 127167 | 132802 | 9.576 | 88759 | 133450 | 6.651 | 98157 | 134091 | 7.320 | 124005 | 134735 | 9.204 |
| **3** | 139986 | 132802 | 10.541 | 138574 | 133450 | 10.384 | 135848 | 134091 | 10.131 | 138683 | 134735 | 10.293 |
| **4** | 139915 | 132802 | 10.536 | 133833 | 133450 | 10.029 | 129351 | 134091 | 9.647 | 128683 | 134735 | 9.551 |
| **5** | 128411 | 132802 | 9.669 | 128598 | 133450 | 9.636 | 125129 | 134091 | 9.332 | 131257 | 134735 | 9.742 |
| **6** | 142182 | 132802 | 10.706 | 127545 | 133450 | 9.558 | 119344 | 134091 | 8.900 | 118740 | 134735 | 8.813 |
| **7** | 129537 | 132802 | 9.754 | 122602 | 133450 | 9.187 | 112647 | 134091 | 8.401 | 117359 | 134735 | 8.710 |
| **8** | 126893 | 132802 | 9.555 | 117221 | 133450 | 8.784 | 115140 | 134091 | 8.587 | 115019 | 134735 | 8.537 |
| **9** | 124152 | 132802 | 9.349 | 112288 | 133450 | 8.414 | 106925 | 134091 | 7.974 | 106188 | 134735 | 7.881 |
| **10** | 109343 | 132802 | 8.234 | 101463 | 133450 | 7.603 | 100392 | 134091 | 7.487 | 100091 | 134735 | 7.429 |
| **11** | 104782 | 132802 | 7.890 | 110414 | 133450 | 8.274 | 110662 | 134091 | 8.253 | 105264 | 134735 | 7.813 |
| **12** | 120341 | 132802 | 9.062 | 105036 | 133450 | 7.871 | 104710 | 134091 | 7.809 | 98306 | 134735 | 7.296 |

|  | **2013** | | | **2014** | | | **2015** | | | **2016** | | |
| --- | --- | --- | --- | --- | --- | --- | --- | --- | --- | --- | --- | --- |
| Month | Disease cases | Population/100,000 | incidence per 100, 000 | Disease cases | Population/100,000 | incidence per 100, 000 | Disease cases | Population/100,000 | incidence per 100, 000 | Disease cases | Population/100,000 | incidence per 100, 000 |
| **1** | 104238 | 135404 | 7.698 | 96067 | 136072 | 7.060 | 95151 | 136782 | 6.956 | 86181 | 137462 | 6.956 |
| **2** | 89582 | 135404 | 6.616 | 88549 | 136072 | 6.508 | 75541 | 136782 | 5.523 | 83527 | 137462 | 5.523 |
| **3** | 125549 | 135404 | 9.272 | 111975 | 136072 | 8.229 | 113242 | 136782 | 8.279 | 114627 | 137462 | 8.279 |
| **4** | 121701 | 135404 | 8.988 | 118849 | 136072 | 8.734 | 107997 | 136782 | 7.896 | 103397 | 137462 | 7.896 |
| **5** | 122172 | 135404 | 9.023 | 106557 | 136072 | 7.831 | 102067 | 136782 | 7.462 | 100500 | 137462 | 7.462 |
| **6** | 106646 | 135404 | 7.876 | 101463 | 136072 | 7.457 | 102388 | 136782 | 7.485 | 94064 | 137462 | 7.485 |
| **7** | 113531 | 135404 | 8.385 | 105782 | 136072 | 7.774 | 102986 | 136782 | 7.529 | 92793 | 137462 | 7.529 |
| **8** | 107051 | 135404 | 7.906 | 97039 | 136072 | 7.131 | 95746 | 136782 | 7.000 | 95924 | 137462 | 7.000 |
| **9** | 105219 | 135404 | 7.771 | 95893 | 136072 | 7.047 | 95081 | 136782 | 6.951 | 89342 | 137462 | 6.951 |
| **10** | 100254 | 135404 | 7.404 | 88991 | 136072 | 6.540 | 86024 | 136782 | 6.289 | 83092 | 137462 | 6.289 |
| **11** | 101595 | 135404 | 7.503 | 86081 | 136072 | 6.326 | 88115 | 136782 | 6.442 | 87963 | 137462 | 6.442 |
| **12** | 100685 | 135404 | 7.436 | 93000 | 136072 | 6.835 | 89818 | 136782 | 6.567 | 89609 | 137462 | 6.567 |

|  | **2017** | | | **2018** | | | **2019** | | | **2020** | | |
| --- | --- | --- | --- | --- | --- | --- | --- | --- | --- | --- | --- | --- |
| Month | Disease cases | Population/100,000 | incidence per 100, 000 | Disease cases | Population/100,000 | incidence per 100, 000 | Disease cases | Population/100,000 | incidence per 100, 000 | Disease cases | Population/10,000 | incidence per 100, 000 |
| **1** | 80911 | 138271 | 5.852 | 96125 | 139008 | 6.915 | 88597 | 139538 | 6.349 | 67682 | 140005 | 4.834 |
| **2** | 92037 | 138271 | 6.656 | 77224 | 139008 | 5.555 | 73096 | 139538 | 5.238 | 44933 | 140005 | 3.209 |
| **3** | 105633 | 138271 | 7.640 | 110124 | 139008 | 7.922 | 97866 | 139538 | 7.014 | 73427 | 140005 | 5.245 |
| **4** | 97296 | 138271 | 7.037 | 100054 | 139008 | 7.198 | 101191 | 139538 | 7.252 | 85684 | 140005 | 6.120 |
| **5** | 101628 | 138271 | 7.350 | 102063 | 139008 | 7.342 | 96106 | 139538 | 6.887 | 83385 | 140005 | 5.956 |
| **6** | 99001 | 138271 | 7.160 | 91603 | 139008 | 6.590 | 99555 | 139538 | 7.135 | 849520 | 140005 | 6.068 |
| **7** | 96471 | 138271 | 6.977 | 95338 | 139008 | 6.858 | 93318 | 139538 | 6.688 | 67682 | 140005 | 5.936 |
| **8** | 100076 | 138271 | 7.238 | 94232 | 139008 | 6.779 | 84304 | 139538 | 6.042 | 44933 | 140005 | 5.459 |
| **9** | 92494 | 138271 | 6.689 | 88302 | 139008 | 6.352 | 80973 | 139538 | 5.803 | 73427 | 140005 | 5.386 |
| **10** | 81554 | 138271 | 5.898 | 84680 | 139008 | 6.092 | 75123 | 139538 | 5.384 | 85684 | 140005 | 4.846 |
| **11** | 89976 | 138271 | 6.507 | 87709 | 139008 | 6.310 | 73000 | 139538 | 5.232 | 83385 | 140005 | 4.974 |
| **12** | 87630 | 138271 | 6.338 | 83205 | 139008 | 5.986 | 71631 | 139538 | 5.133 | 84952 | 140005 | 4.578 |
